# *Drosophila* as a Model Organism in Host–Pathogen Interaction Studies

**DOI:** 10.3389/fcimb.2020.00214

**Published:** 2020-06-23

**Authors:** Salma Younes, Asma Al-Sulaiti, Elham Abdulwahab Ahmed Nasser, Hoda Najjar, Layla Kamareddine

**Affiliations:** Biomedical Sciences Department, College of Health Sciences, QU Health, Qatar University, Doha, Qatar

**Keywords:** *Drosophila*, host–pathogen interactions, host defense factors, pathogen virulence factors, disease control, disease progression

## Abstract

Owing to the genetic similarities and conserved pathways between a fruit fly and mammals, the use of the *Drosophila* model as a platform to unveil novel mechanisms of infection and disease progression has been justified and widely instigated. Gaining proper insight into host–pathogen interactions and identifying chief factors involved in host defense and pathogen virulence in *Drosophila* serves as a foundation to establish novel strategies for infectious disease prevention and control in higher organisms, including humans.

## Introduction

*Drosophila*, a chief tool in contemporary genetic studies, became one of the most powerful model organisms widely used in scientific explorations. The versatility, low cost, short life cycle, well-characterized genome, and feasibility of genetic manipulation made the fruit fly an indispensable model organism for basic research. The modern era of *Drosophila* research initially took off when the fly was deployed in developmental biology, particularly in fly embryo studies to identify novel genes involved in development (Nusslein-Volhard and Wieschaus, 1980). Further studies conducted in *Drosophila* have contributed to novel groundbreaking findings that allowed the identification of fundamental components of different pathways conserved between the fruit fly and higher mammalian organisms, including humans. Recently, *Drosophila* gained great popularity in host–pathogen interaction and infectious disease control studies due to several reasons, many of which were attributed to evolutionary conserved features in both *Drosophila* and vertebrates including innate immune cascades, signal transduction pathways, and transcriptional regulators. The fruit fly surprisingly serves as a host for a diversity of pathogens and could be readily infected with these pathogens naturally or in an experimental setting. The existence of a wide array of molecular and genetic tools that allow gene manipulation in specific cells/tissues in the fly also favors its use in host–pathogen interaction studies. Genetic and genome-wide RNAi screens in either intact flies or cell lines have identified a wide array of host effector molecules and pathways involved in host defense against invading pathogens. Reciprocally, flies can be used to screen for pathogen-virulence factors. The fly's GAL4-UAS transactivation system (Brand and Perrimon, 1993) allows the direct expression of transgenes encoding host or pathogen proteins in a cell-type-specific manner *in vivo*. Also, the fly's LexA transcriptional system, allows combinatorial gene expression in a distinct or overlapping fashion *in vivo* (Pfeiffer et al., 2010; Yagi et al., 2010), opening up for the feasibility of conducting epistasis analysis and revealing a role of specific genes in regulating cellular processes and pathways. Such experiments are difficult to be conducted in higher model organisms including mammals, advocating the use of *Drosophila* in host–pathogen interaction studies. Like all invertebrates, *Drosophila* lacks an adaptive immune response and relies exclusively on innate immunity with both its humoral and cellular arms to fight off invading pathogens. These innate immune responses mainly include production of antimicrobial peptides (AMPs) and anti-pathogenic factors through core signaling pathways (Toll, IMD, and JAK/STAT), anti-viral response through the RNA interference (RNAi) pathway, and pathogen immobilization through phagocytosis, encapsulation, and melanization (Agaisse and Perrimon, 2004; Akira et al., 2006; Govind, 2008). In this review, we provide an overview of the use of *Drosophila* in host–pathogen interaction studies and highlight the role of the fly's innate immune system in pathogen control. We also recapitulate a broad spectrum of host defense and pathogen virulence factors identified in *Drosophila*-pathogen studies and involved in microbial control and disease progression.

## Host Defense Factors

**Figure 1:**
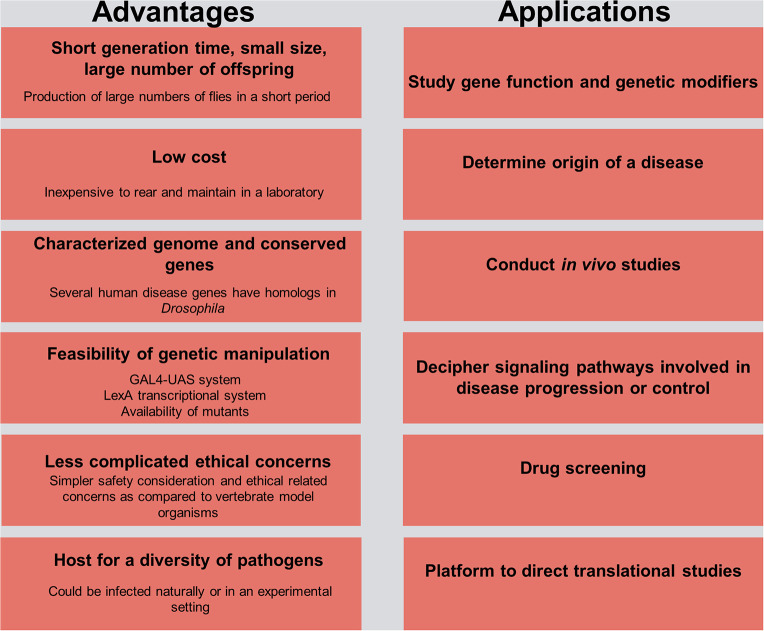
Advantages and practical applications in *Drosophila* for host–pathogen interaction studies. The left side of the figure delineates the advantages of using the fruit fly model organism in research, and the right side outlines its use as a platform for understanding the etiology of a disease and the potential means of controlling it.

*Drosophila* is considered a significant model organism in studying host–pathogen interactions (Figure 1). The establishment of the *D. melanogaster* whole genome sequence in 2000 (Adams et al., 2000) paved the way for adapting existing high-throughput RNAi screening methodologies in *Drosophila* cell lines to study gene function and identify specific gene targets and immune-associated components and modulators (Ueda, 2001; Kiger et al., 2003). Combining the findings of high-throughput RNAi screens with classical genetic methods and *in vivo* fly studies enabled the identification of humoral and cell-mediated host defense factors against a wide array of intracellular and extracellular pathogens (Cherry, 2008; Bier and Guichard, 2012).

### Humoral Host Defense

Humoral innate immune responses in *Drosophila* mainly include production of AMPs and anti-pathogenic factors through Toll, IMD, and JAK/STAT signaling pathways. The primarily role attributed to the Toll pathway was its involvement in *Drosophila* embryonic development (Nusslein-Volhard and Wieschaus, 1980). In 1995, Hultmark et al. introduced Toll (Toll-1) as a potent immune activator in fruit fly cell lines (Rosetto et al., 1995). Since then, the Toll pathway was shown to be implicated in immune defense against an array of pathogens. Unlike the mammalian Toll pathway, the activation of *Drosophila* Toll signaling is not initiated by direct interaction with microbial determinants, but rather by the cleaved form of *spätzle*, a cytokine-like molecule that is thought to be processed by secreted serine proteases (SPs) and spätzle-processing enzyme (SPE). SPs and SPE are regulated by several pathogen recognition receptors (PRRs) including peptidoglycan recognition protein SA (PGRP-SA), PGRP-SD, Gram-negative binding protein 1 (GNBP1), and GNBP3 (Gottar et al., 2006). To avoid exaggerated immunity, the activation of the Toll pathway is generally tightly regulated. Upregulation of Spn1, a member of the serpin superfamily protease inhibitors located upstream of SPE, for example, contributes to the Toll pathway inactivation and to a downregulation in the expression of AMPs, mainly *Drosomycin*. Fungal-infected *Spn1* null mutants exhibit an up-regulation in *Drosomycin* (Fullaondo et al., 2011). ModSP, a modular serine protease, activates the Toll pathway to culminate in AMP production. *ModSP* mutant flies challenged with either gram-positive bacteria (*Enterococcus faecalis* or *Listeria monocytogenes*) or fungal species (*Candida albicans*) succumb to death-associated reduction in AMP gene expression (Buchon et al., 2009a). In addition to its well-defined role against fungal and gram-positive bacteria, Oh et al. reported a role of the Toll pathway in defense against acid-fast mycobacteria. *Mycobacterium abscessus*, a non-tuberculous mycobacteria in humans, colonizes the gut of *D. melanogaster* and induces predominant expression of *Drosomycin* upon Toll pathway activation (Oh et al., 2013). Strikingly, Gottar et al. identified a pathway that acts jointly with GNBP3 to activate the Toll pathway upon fungal infection. PR1, a *C. albicans* virulence factor, activates Toll signaling by promoting the proteolytic cleavage and maturation of the Persephone protease (PSH). This finding indicates that the detection of fungal infection in *Drosophila* is dependent on both the recognition of foreign fungal invariant patterns and on tracking the consequence of virulence elements on the infected host (Gottar et al., 2006). Interestingly, and although both GNBP3 and PSH-dependent pathway are also required for Toll pathway activation upon *Candida glabrata* infection, only GNBP3 mutants are susceptible to *Candida glabrata* infection, implicating that the downstream effector mechanisms like AMP production and melanization activated against different fungal infections may not be the same (Chamilos et al., 2010; Quintin et al., 2013). Several studies have also employed *D. melanogaster* as a model organism to characterize anti-viral Toll immunity. The Toll pathway was shown to play a role in efficiently inhibiting *Drosophila* X viral (DXV) replication. Interestingly, the levels of *Drosophila* AMP genes induced in response to DXV infection were similar to those reported during *Escherichia coli* infection (Zambon et al., 2005). Extracellular virions, which were first discovered in *Drosophila*, and currently in metazoans, are also recognized by Toll-like receptors located on cell surfaces and inside endo-lysosomal compartments (Medzhitov, 2001).

Recently, the impact of post-translational modifications on modulating Toll signaling has been also studied in fruit flies. Such modifications were shown to change the localization and trafficking of the protein in a cell, enhance or inhibit the protein activity, and/or alter the protein's ability to bind to protein signaling partners. The *Drosophila* Ubc9/Lwr enzyme, for example, affects Toll signaling by stimulating the sumoylation of the Dorsal transcription factor (Schmidt, 2014). Likewise, β-arrestin Kurtz (Krz) regulates Toll signaling via protein sumoylation by interacting with the SUMO protease Ulp1. *Krz* or *Ulp1 Drosophila* larval mutants exhibit inflammation-like phenotypes characterized by elevation in lamellocyte production, formation of melanotic tumors, accumulation of transcriptional effectors (Dorsal and Dif) of the Toll pathway, and increased expression of anti-microbial peptides (*Drosomycin*). Interestingly, loss of function of these two genes reveal a dose-dependent sensitive and synergistic response, suggesting that they belong to the same signaling pathway (Anjum et al., 2013). Moreover, Pellinos, a family of E3 ubiquitin ligases, were shown to also regulate Toll signaling by catalyzing the K63-linked polyubiquitination of Pelle, an IL-1 receptor-associated kinase homolog in *Drosophila* (Medvedev et al., 2015). Genome-wide screening studies of the Toll pathway also identified novel immune-associated components and regulators including the Deformed Epidermal Auto-regulatory Factor 1 (DEAF1) transcription factor as an essential component for the expression of the Toll target AMP *Drosomycin* (Kuttenkeuler et al., 2010).

Similar to the Toll pathway, the *Drosophila* IMD pathway, which is mainly directed against gram-negative pathogens, plays a fundamental role in humoral immunity through AMP production and pathogen clearance. IMD mutant flies, for example, are sensitive to *Vibrio cholerae* infection (Wang et al., 2013; Kamareddine et al., 2018a), while those with a gain-of-function mutation exhibit resistance, plausibly by lowering the virulence effect of the cholera toxin via increasing the rate of intestinal stem cell division (Wang et al., 2013). *Dredd*^*D*55^ IMD mutant flies infected with *Xenorhabdus nematophila* and *Photorhabdus luminescens* nemato-bacterial composites also fail to survive infection compared to *Dif*^1^ Toll mutants and wild-type infected flies, albeit the 24 h priming with non-pathogenic *E. coli* prior to *X. nematophila* and *P. luminescens* infection. These findings advocate the notion that *X. nematophila* and *P. luminescens* pathogens target components of the IMD pathway, despite AMPs synthesis triggered by the nemato-bacterial composite infection (Aymeric et al., 2010). Apart from its well-defined role against gram-negative bacteria, recent studies have also highlighted a role of IMD signaling in defense against fungal and gram-positive bacterial infection (De Gregorio et al., 2002a; Hedengren-Olcott et al., 2004; Pham et al., 2007; Dionne and Schneider, 2008; Costa et al., 2009). Interestingly, non-canonical AMP-independent IMD immunity have been also shown to be crucial in the *Drosophila* gut defense system. Hori et al. reported that IMD mutant flies are sucseptible to *Staphylococcus aureus* oral infections and revealed a role of the IMD pathway in clearance of *S. aureus* from the fly gut (Hori et al., 2018). In alliance with this distinctive role in gut immunity, the IMD pathway was also shown to control gut homeostatic balance in a microbiota-dependent–infection-independent context. The gut flora, which induces IMD signaling activation, significantly affects the midgut transcriptome and promotes the expression of key genes involved in host physiology. A study by Kamareddine et al. revealed that IMD signaling in enteroendocrine cells activated by the intestinal microbiota acetate metabolite regulates the expression of the tachykinin peptide hormone, promoting metabolic homeostasis in the host. Both germ-free flies and IMD mutant flies were shown to behave similarly by exhibiting developmental retardation, disrupted lipid metabolism, and a status of inactive insulin signaling (Kamareddine et al., 2018a). Owing to the fact that humoral immunity in *Drosophila* is chiefly mediated by AMP production by fat body cells, particular attention has been also given to our understanding of IMD signaling in the fat body. A study by Tsichritzis et al. (2007) revealed that the deubiquitinase Cylindromatosis (CYLD) inhibits NF-κB signaling and downregulates the IMD response. Although CYLD mutant flies exhibit an increase in AMP expression, particularly those with prior infections, yet these mutants succumb to death significantly faster than controls upon *E. coli* infection. Although the target of CYLD in the IMD pathway remains uncharacterized, this poor survival rate of CYLD-deficient flies could be attributed to an alteration in the structure and function of fat body cells, as CYLD regulates homeostatic balance in these cells. Interestingly, several factors that affect physiology and development in a host also affect Toll and IMD signaling through manipulating fat body maturation. The induction of *Diptericin* expression in larvae, for example, is affected by age and is dependent on the presence of the ecdysone molting hormone. A mutation affecting the metabolism of ecdysone could indirectly affect the immune status of a host (Meister and Richards, 1996; Ligoxygakis et al., 2002a). It is worth noting here that signaling mechanisms between the gut and the fat body contribute to the regulation of systemic immune responses in the host (Lemaitre and Hoffmann, 2007). Upon oral infection, *Ecc15* and *P. entomophila* can colonize and multiply in the fly gut, triggering strong systemic immunity, without a need for those bacterial species to cross the wall of the gut (Vodovar et al., 2005; Acosta Muniz et al., 2007).

Since the IMD pathway is similar to the mammalian tumor necrosis factor receptor (TNFR) pathway (Leulier et al., 2000; Costa et al., 2009), which plays a critical role in infectious disease control particularly against viral infections (Herbein and O'Brien, 2000), several studies deployed *Drosophila* as a model organism to gain further insight into the role of IMD signaling in anti-viral immunity. The cricket paralysis virus (*CrPV*), an RNA virus that infects a wide range of insect hosts, displays increased virulence with higher viral loads in IMD mutant flies (Costa et al., 2009). Interestingly, IMD signaling-mediated anti-*CrPV* immunity seems to be also AMP independent. Similar to *CrPV* infection, sindbis viral replication increases in IMD mutant flies (Avadhanula et al., 2009). Moreover, knocking down the peptidoglycan recognition protein-LC (PGRP-LC), a membrane associated IMD pathway receptor, in *Drosophila* S2 cells also promotes an increase in the genome copy number of the sigma virus and causes a significant up-regulation in the expression of the *L* gene compared to other viral genes (Liao et al., 2019). An anti-microbial RNAi signaling screen performed by Foley et al. publicized different classes of negative and positive gene regulators of IMD signaling including those that enhance response to peptidoglycan stimulation (46 EDRi genes), and others that constitutively activate NF-kB in the absence of LPS induction (26 CDRi genes) (Blandin et al., 2004). Further screens identified additional IMD positive regulators including Iap2 and TAB (Garver et al., 2006; Kawai and Akira, 2006). Similar to the studies that have been conducted on the Toll pathway, the impact of post-translational modifications on regulating IMD signaling has been also recently deliberated in fruit flies. SP36/Scny was shown to negatively regulate IMD signaling transduction by hydrolyzing UbK63, a key player in IMD ubiquitination (Thevenon et al., 2009). Similarly, USPs were also shown to regulate IMD immune signaling. USP2, for instance, deubiquitinates Imd, promoting its degradation (Engel et al., 2014).

**Figure 2:**
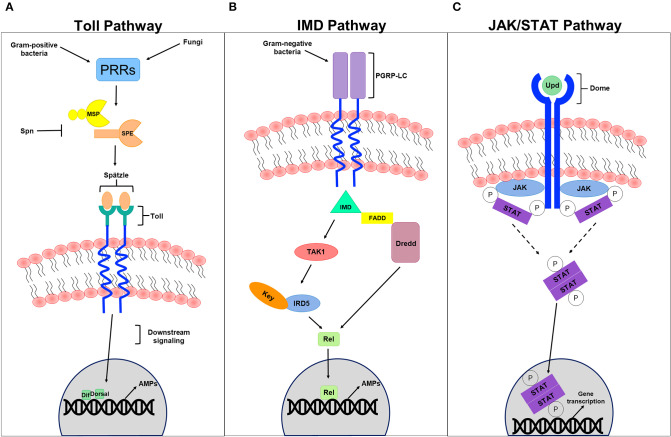
Humoral innate immune signaling pathways. **(A)** Represents a schematic diagram of the Toll pathway. Gram-positive bacteria and fungi recognized by pathogen recognition receptors (PRRs) trigger the activation of this pathway. Modular serine protease (MSP) and spätzle-processing enzyme (SPE), which are regulated by several PRRs are thought to process the cleavage of the *spätzle* ligand into a mature *spätzle* that binds to the Toll receptor, initiating downstream signaling pathway that culminates in the translocation of the NFB-like transcription factors Dif and/or Dorsal into the nucleus, promoting the expression to antimicrobial peptides (AMPs) in response to infection. Serpin (Spn) tightly regulates the primary steps of this pathway to avoid exaggerated immunity. **(B)** Represents a schematic diagram of the IMD pathway. Gram-negative bacteria recognized by receptors of the IMD pathways like the peptidoglycan recognition protein-LC (PGRP-LC) trigger the pathway activation, promoting the formation of the IMD, FADD, and Dredd (caspase 8 homolog) complex. This in turn activates Dredd, which is thought to be involved in the cleavage of the NFB-like transcription factors Relish (Rel). This formed complex also activates Tak1 (MAP3 kinase) and the IKK complex (IRD5 and key) to phosphorylate Rel. Once translocated into the nucleus, Rel promotes the expression to AMPs in response to invading pathogens. **(C)** Represents a schematic diagram of the JAK/STAT pathway. The UPD ligand binds to the DOME receptor leading to its activation. The phosphorylation of JAK and DOME create docking puts for STATs recruited to the formed complex. STATs themselves become phosphorylated generating an active dimer that translocates to the nucleus, promoting effector gene expression.

The JAK/STAT signaling pathway, which controls various biological processes and tissue hemostasis in both mammals and invertebrates, also contributes to humoral immunity in a host. It is mainly activated upon microbial infection and/or cellular damage induced by stress response/pathogen infection, and culminates in the production of regulatory molecules, anti-viral agents, and anti-bacterial agents including AMPs. Cell damage induced by *Serratia marcescens* and *Erwinia carotovora* infection in *Drosophila* for example induces JAK/STAT signaling (Buchon et al., 2009b; Cronin et al., 2009) and activates a gut-specific defense machinery characterized by the expression of a subset of AMPs including the *Drosomycin-like peptide* (*dro3*). This activation, which is pathogen specific and triggered by cell damage caused by bacterial infection rather than by the bacteria itself (Buchon et al., 2009c), is particularly important in maintaining gut homeostasis by controlling epithelial cell proliferation and renewal in response to bacterial infection (Buchon et al., 2009b; Jiang et al., 2009). In the absence of infection, the indigenous gut flora triggers the expression of *hop*^*Tum*−*l*^ or *upd3*, which is generally adequate to induce intestinal stem cell progeny differentiation and gut regeneration through JAK/STAT and JNK signaling (Buchon et al., 2009b). Moreover, global gene expression analysis of *Drosophila* gut tissues to oral *Erwinia carotovora* infection revealed an important contribution of IMD and JAK/STAT pathways, but not the Toll pathway, to the regulation of gut immune responses (Buchon et al., 2009c). Although the intricate contribution of JAK/STAT signaling to cellular immunity has not been fully understood, it has been thought to be involved in cellular responses like hemocyte proliferation and differentiation (Agaisse and Perrimon, 2004). Recently, Yang et al., reported a role of JAK/STAT signaling in parasitoid egg wasp encapsulation in infected *Drosophila* larvae (Yang et al., 2015). Several studies addressing the role of JAK/STAT pathway in anti-viral immunity also revealed that the expression of “traditional” JAK/STAT pathway target genes including *upd2, upd3*, and *TotM*, is induced by many viral species including vesicular stomatitis virus, Flock House virus, and *Drosophila* X virus (Kemp et al., 2013; Myllymaki and Ramet, 2014). Likewise, the *Drosophila* C virus infection triggers the expression of several genes like *virus-induced RNA-1 (vir-1)*. Many of these induced genes enclose STAT binding sites in their promoter regions, and their activation is therefore dependent on JAK/STAT signaling. The JAK tyrosine kinase Hopscotch (Hop) was also shown to be involved in controlling *Drosophila* C virus loads and to participate in inducing the expression of some virus-regulated genes. Deficiencies in JAK/STAT signaling increases *Drosophila* C virus load and exhibits high mortality rates in infected flies (Dostert et al., 2005). Although double-stranded RNA (dsRNA) itself does not induce viral response in *Drosophila*, recent studies have shown that recognizing virus-derived dsRNA through the amino terminal DExD/H-box helicase domain of Dicer-2 promotes the expression of the vago-secreted protein (Paradkar et al., 2012) that plays an antiviral role against *Drosophila* C virus infection (Paradkar et al., 2012). Interestingly, vago seems to induce the JAK/STAT pathway through a Dome-independent mechanism, signifying the existence of an alternative receptor that is yet to be determined. This finding provides a conceivable role of vago in connecting both RNAi and JAK/STAT signaling pathways, suggesting that vago, which is thought to be insect specific, could serve as a cytokine and functionally relate to the mammalian interferon system (Paradkar et al., 2012). By comparing RNA interference (detailed in the section below) with JAK/STAT anti-viral immunity, however, RNAi interference epitomizes an effectual antiviral machinery that operates against an array of RNA and DNA viruses, unlike the antiviral contribution of JAK/STAT signaling, which seems to be more species specific (Kemp et al., 2013) (Figure 2).

### Cell-Mediated Host Defense

Phagocytosis, which is involved in ingesting apoptotic debris and destroying foreign pathogens by hemocytes (plasmatocytes, crystal cells, and lamellocytes), represents a fundamental mean of maintaining tissue homeostasis (Lemaitre and Hoffmann, 2007). Various receptors and key players chiefly involved in the phagocytic process have been identified in *Drosophila*. Pearson et al. revealed that the *Drosophila* scavenger receptor C1 (SR-CI), which has a broad polyanionic ligand-binding specificity similar to the mammalian class A macrophage-specific scavenger receptor (SR-A), exhibits great affinity and saturable binding of ^125^I-labeled acetylated low-density lipoprotein when expressed in mammalian cells (Pearson et al., 1995). Cuttell, et al. highlighted a previously uncharacterized role of the CED1/6/7 pathway in phagocytosis, by demonstrating that Draper (a CED-1homolog that belongs to the CED1/6/7 pathway)-mediated phagocytosis requires the *Drosophila* Junctophilin protein, Undertaker (UTA), and is linked to Ca^2+^ homeostasis (Cuttell et al., 2008). Additionally, Kocks et al. identified a role of Eater, an EGF-like repeat transmembrane receptor of the Nimrod family present on *Drosophila* hemocytes, in bacterial phagocytosis (Kocks et al., 2005). Likewise, Bretscher et al., uncovered the contribution of Eater in hemocyte localization, attachment, and adhesion, and in efficient phagocytosis of gram-positive (*Staphylococcus aureus, Staphylococcus epidermidis, Micrococcus luteus*), but not gram-negative (*Escherichia coli* and *Serratia marcescens*) bacteria (Bretscher et al., 2015). The intergin βν phagocytic receptor was also shown to be involved in defense against septic but not oral *S. aureus* infection in *Drososphila* (Shiratsuchi et al., 2012). Studies in *Drosophila* S2 cells in turn identified a role of PGRP-LC in phagocytosis of gram-negative (*E. coli*), but not gram-positive bacteria (Ramet et al., 2002). Apart from its scavenger function, PGRP-SC1 was also shown to act as an opsonin, and therefore, contribute to bacterial phagocytosis (Garver et al., 2006). Several thioester proteins (TEPs) identified in different insect species including *Anopheles gambiae*, also act as a bona fide opsonin to promote gram-positive and gram-negative bacterial phagocytosis (Levashina et al., 2001). In *Drosophila*, functional data publicized a role of several fruit fly TEPs, including TEP2, TEP3, and TEP6 in binding to several pathogens including *E. coli, S. aureus*, and *C. albicans*, respectively (Stroschein-Stevenson et al., 2006). Interestingly, Croquemort (CRQ), a CD36-related receptor that is exclusively expressed on macrophages in *Drosophila* embryo, was shown to be required for effectual phagocytosis of apoptotic corpses, but is not necessary for bacterial engulfment (Franc et al., 1999). Several screens identified cellular mediators of phagocytosis. Among those genes are four transcription factors, one of which encodes the GATA-factor Serpent, a chief regulator of hematopoesis in flies. Complimentary expression profile studies identified 45 genes, including the SR-C1 scavenger receptor gene that is down-regulated upon Serpent depletion (Meister and Tuschl, 2004; Haasnoot and Berkhout, 2006). RNAi against these Serpent-dependent genes further identified Eater and Nimrod phagocytic receptors (Miyano-Kurosaki and Takaku, 2006). Given that various classes of entry receptors plausibly facilitate the uptake of different microbes, although overlying and repetitive specificities do exist occasionally, many screen studies are usually done following specific microbial infections. Stroschein-Stevenson et al. identified 184 genes essential for efficient fungal uptake using *Candida*-infected phagocytic S2 cells. Among those genes is the macroglobulin-related protein (Mcr), which specifically opsonize *Candida*, unlike TEP2 and TEP3 that are needed for opsonization and efficient uptake of *E. coli* and *S. aureus*, respectively (Stroschein-Stevenson et al., 2006). Another screen following *Mycobacteria fortuitum* infection revealed 54 genes including the novel class B scavenger receptor peste (Li et al., 2002) that is essential for *Mycobacteria fortuitum* and *Listeria monocytogenes*, but not for *E. coli* and *S. aureus* uptake in S2 cells (Galiana-Arnoux et al., 2006). Genome-wide RNA interference screening was put forth to introduce host factors that block intracellular bacterial pathogenesis using cells. Interestingly, comparative studies of host defense genes involved in hindering bacterial pathogenesis revealed that some host factors have general inhibitory roles in intracellular pathogenesis, while others specifically affect the mechanistic ability of certain bacterial species to access the host (Agaisse et al., 2005). Rab7, CG8743, and the ESCRT machinery, for instance, represent unique vulnerability factors of the host cell, as manipulating any of these factors alone no longer constrains the growth of the non-pathogen *Mycobacterium smegmatis* in *Drosophila* (Yang et al., 2015). A similar study on *Mycobacterium marinum* also identified the lysosomal enzyme beta-hexosaminidase as an imperative factor in modulating mycobacterial growth. Remarkably, this bactericidal activity of β-hexosaminidase seems to be *Mycobacterium marinum* specific, as it is not involved in constraining the growth of other bacterial species like *Salmonella typhimurium* and *Listeria monocytogenes* (Koo et al., 2008).

**Figure 3:**
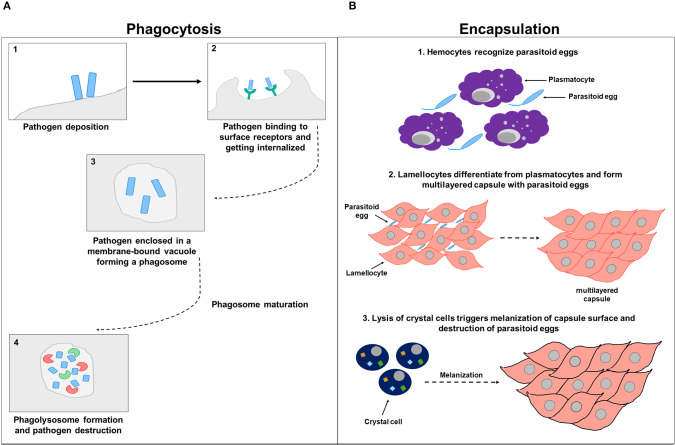
Cell-mediated immunity. **(A)** Represents a schematic diagram of phagocytosis. After the pathogen deposits on the host cell surface (1), it binds to phagocytic receptors and gets internalized (2) and enclosed in a membrane-bound vacuole forming a phagosome (3). The phagosome undergoes subsequent phases of maturation before eventually forming a phagolysosome that contains factors including DNases and proteases involved in pathogen destruction (4). **(B)** Represents a schematic diagram of encapsulation. In the first stage of encapsulation, hemocytes recognize parasitoid eggs as foreign invaders, triggering downstream signaling (1). In the second stage of encapsulation, hemocytes increase in numbers and lamellocytes differentiated from plasmatocytes and attach to parasitoid eggs and to each other, forming a multilayered capsule (2). In the third stage of encapsulation, crystal cells are involved and synthesize enzymes needed for melanization. Parasitoid eggs get sheathed, immobilized by the deposited melanin, and destroyed within the capsule either by direct asphyxiation or by the release of superoxide anions or hydroxyl radicals (3).

Encapsulation is a another cellular response that is devoted to eliminate pathogens by forming hemocytic capsules around foreign bodies that are outsized to be phagocytozed (Kounatidis and Ligoxygakis, 2012). In *Drosophila*, cellular encapsulation happens in three stages. During the first stage, hemocytes, plausibly through their surface receptors, primarily recognize the parasitoid egg as a non-self. This recognition further promotes changes in the hemocyte cell surface membrane, exposing hidden molecules and presumably triggering downstream signaling (Nappi et al., 1991, 2000). During the second stage of encapsulation, the number of circulating hemocytes increases for a short term, and lamellocytes differentiate from plasmatocytes (Rizki and Rizki, 1990). Plasmatocytes account for more than 90% of all mature larval hemocytes and are involved in the phagocytic elimination of pathogenic microorganisms and dead cells (Lemaitre and Hoffmann, 2007). Activated lamellocytes, which are only present in larvae and whose expression is mainly induced upon infection, traffic to the parasitoid egg, flatten, and attach to the egg and to each other, creating a multilayered capsule (Strand and Pech, 1995). Lamellocytes are particularly involved in encapsulating and neutralizing invading pathogens that are too large to be up-taken by phagocytosis (Lemaitre and Hoffmann, 2007). The third stage of encapsulation involves crystal cells that account for 5% of the larval hemocytes. These cells function as storage sites for pro-phenoloxidase (PPO) and are therefore involved in the melanotic defensive response. Lysis of crystal cells triggers the melanization of the capsule surface (Strand and Pech, 1995; Fellowes and Godfray, 2000; Lemaitre and Hoffmann, 2007). Within the capsule, the encapsulated parasitoid egg gets killed by either direct asphyxiation (Salt, 1970) or by the release of superoxide anions or hydroxyl radicals from the capsule content (Nappi et al., 1995, 2000; Nappi and Vass, 1998) (Figure 3).

Melanization is another prominent immune response in insects characterized by melanin synthesis and deposition around intruding microorganisms (Christensen et al., 2005). Melanization is also involved in wound healing, phagocytosis, blood coagulation, and AMP expression in arthropods (Ashida and Brey, 1995; Söderhäll and Cerenius, 1998; Cerenius et al., 2008). The melanotic reaction, which is generally induced by either a pathogenic infection or tissue injury, culminates in the proteolytic cleavage of inactive PPO to active phenol oxidase (PO), the chief enzyme in melanin biogenesis (Cerenius et al., 2008). To avoid the production of excessive intermediates that are toxic to the host, the activation of melanization is normally tightly regulated (De Gregorio et al., 2002b; Ligoxygakis et al., 2002b; Scherfer et al., 2008; Tang et al., 2008). In *Drosophila*, genetic studies identified melanization regulators including serine proteases and serpin proteins. MP1 and MP2/sp7/PAE1 clip proteases, for example, positively regulate melanization. Silencing either MP1 or MP2 inhibit PO activation upon pathogenic infection (De Gregorio et al., 2002b; Ligoxygakis et al., 2002b; Castillejo-Lopez and Hacker, 2005; Scherfer et al., 2008; Tang et al., 2008). Several studies also highlighted a role of PGRPs in inducing melanization. The proPO cascade in *Drosophila* larvae is induced by a forced expression of PGRP-LE, independent of infection. Consistent with this, PGRP-LE is required for infection (*E. coli*)-induced melanization (Takehana et al., 2002, 2004). Likewise, PGRP-LC regulates melanization in *Drosophila* (Schmidt et al., 2007). Although melanization is considered an integral component in insect immunity, evidence of direct killing through quinone synthesis and melanin production has been reported in a few studies in insect species only. In *Anopheles gamabiae*, melanization was shown to retard *Beauveria bassiana* growth and dissemination (Yassine et al., 2012). In *Manduca sexta*, however, 60–94% killing of a broad spectrum of gram-negative bacterial species including *Klebsiella pneumoniae, Escherichia coli, Pseudomonas aeruginosa*, and *Salmonella typhimurium*, and 52–99% killing of gram-positive bacterial species including *Staphylococcus aureus, Bacillus subtilis, Bacillus cereus*, and *Micrococcus luteus* was reported in an active melanotic milieu (Zhao et al., 2007). Similarly, a recent study in *D. melanogaster* publicitized a novel role of melanization in anti-nematode immunity (Cooper et al., 2019). It is worth noting here that some host–pathogen interactions are “genotype by genotype” driven. *Drosophila melanogaster*'s melanotic and complement-like immunity, for example, vary extensively against the parasitoid wasp *Leptopilina boulardi*. PO activity is predominantly affected by the host genotype, while TEP1 upregulation is controlled by the parasite genotype itself. Lamellocyte differentiation, on the other hand, depends on the specific combination of both the host and parasite genotypes (Leitão et al., 2019).

## RNA Interference

**Figure 4:**
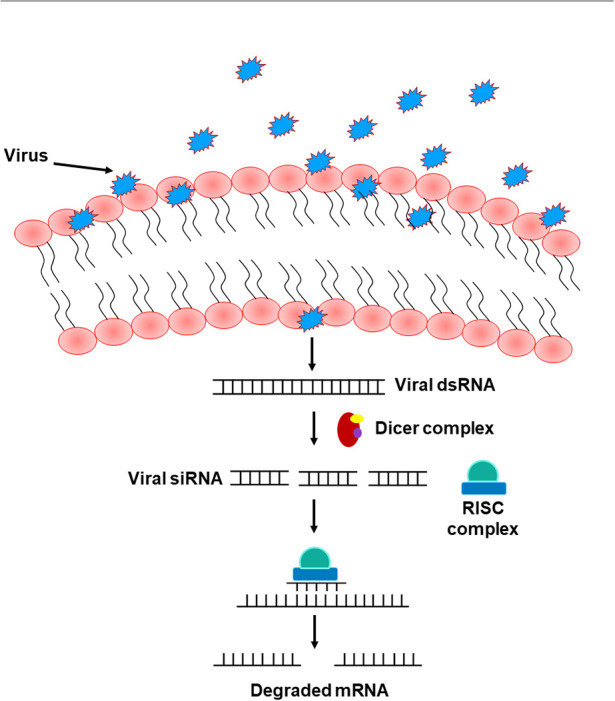
The RNA interference pathway. Upon entry into the cells, viruses shed their shielding external coat, uncovering their RNA, and forming dsRNA. This formed dsRNA gets recognized by the Dicer complex and processed to form viral siRNA. A single strand of this siRNA gets incorporated into the RISC complex and act as a template to recognize complementary mRNA, resulting in mRNA cleavage and therefore silencing of viral RNA.

The RNA interference (RNAi) pathway, which suppresses gene expression through targeted RNA degradation, embodies an ancient mechanism of anti-viral immunity in plants, nematodes, and arthropods including *Drosophila* (Hamilton and Baulcombe, 1999; Li et al., 2002; Lu et al., 2005; Wilkins et al., 2005; Cherry and Silverman, 2006; Wang et al., 2006; Zambon et al., 2006; Saleh et al., 2009; Karlikow et al., 2014). This pathway emerges in two major phases including the “initiation” and the “execution” phase. Either endogenous (short hairpin RNAs manufactured by the genome, perversely expressed trans-genes, and transposons) or exogenous sources (naturally occurring or experimentally made dsRNA) can introduce dsRNA to initiate RNAi (Hannon, 2002; Zambon et al., 2006). dsRNA are recognized and cleaved by Dicer molecules to form small RNAs (Hammond et al., 2000; Blaszczyk et al., 2001; Zambon et al., 2006) that get integrated into the RNA-induced silencing complex (RISC), denoting the execution phase of the RNAi pathway (Blaszczyk et al., 2001; Nykanen et al., 2001; Zambon et al., 2006). Unlike mammals that have only one *Dicer* gene, and which is difficult to study, flies possess two genes, *Dicer1* and *Dicer2*, that are required for processing miRNA precursors from pre-miRNA and siRNA precursors from long dsRNA, respectively (Robles-Sikisaka et al., 2001). The single strand of either miRNA or siRNA integrated into the RISC complex acts as a platform for RISC to recognize complementary messenger RNA (mRNA) transcript. Upon recognition, Argonaute, one of the proteins in RISC, activates and cleaves the mRNA, inhibiting antiviral functions and suppressing viral expression (Karlikow et al., 2014) (Figure 4). Other existing, yet poorly identified, RNAi pathways include the PIWI-interacting RNA (piRNA) pathway that shields host cells from endogenous mobile genetic elements (Buchon et al., 2014). Several studies in *Drosophila* reported that loss-of-function mutations in essential RNAi pathway genes increase host vulnerability to viral infection (Zambon et al., 2006; Aliyari et al., 2008; Buchon et al., 2014). In mammals, other antiviral defense strategies, including protein sensors that recognize viral dsRNA motifs, have been identified. Among these sensors are the DEAD-box helicases RIG-I (Retinoic acid-inducible gene I) and MDA5 (Melanoma Differentiation-Associated protein 5), together known as RIG-I-like receptors (RLRs). Upon recognizing viral nucleic acid during the primary viral infection stages, these sensors induce the expression of type 1 interferons (IFNα and IFNβ) and other pro-inflammatory cytokines (Song and Rossi, 2017; van der Veen et al., 2018; Brisse and Ly, 2019). Interestingly, studies in *Drosophila* showed that Dicer-2 closely resembles the mammalian RLRs, not only by cleaving dsRNA into siRNA, but also by activating the transcription of antiviral effectors proteins (Deddouche et al., 2008). Genetic screening in the fruit fly revealed additional antiviral roles of DEAD-box helicase. DDX17 (known as Rm62), for example, exhibits antiviral activity against arthropod-borne bunyaviruses (Deddouche et al., 2008). In addition to these nucleic acid-elicited responses, some viruses can be directly recognized by Toll-7, which promotes the activation of antiviral autophagy in an AKT pathway-dependent manner through phosphoinositide 3-kinase (PI3K) and target of rapamycin (Tor) (Buchon et al., 2014). Likewise, other studies in *Drosophila* also revealed a direct antiviral role of autophagy against the vesicular stomatitis virus, initiated by the pathogen surface glycoprotein VSVG (Shelly et al., 2009). Sabin et al. identified Ars2 (CG7843) as a key element of *Drosophila* antiviral immunity using an RNAi library and demonstrated that a loss of Ars2 function in either cells or flies promotes vulnerability to RNA viruses. In addition to its antiviral characteristic, Ars2 was shown to modulate Dcr-2 activity *in vitro* by physically interacting with it. It was also shown to play an essential role in siRNA- and miRNA-mediated silencing. This crucial role of Ars2 in these small RNA pathways delivers novel insight into the biogenesis of small RNAs, a platform that could be extended to other systems (Sabin et al., 2009). Similarly, unrecognized host genes imperative for the influenza viral replication have been identified using genome-wide RNAi screens in *Drosophila*. Three of these identified genes have corresponding homologs in humans (ATP6V0D1, COX6A1, and NXF1). When tested in human HEK 293 cells, these genes were shown to be involved in the replication of H5N1 and H1N1 influenza A viruses, but not in vaccinia nor in vesicular stomatitis viral replication (Hao et al., 2008). The natural resistance-associated macrophage (NRAMP), a divalent metal ion transporter and a cell surface molecule expressed on *Drosophila* cells and required for binding and entry of sindbis virus to host cells, was also identified using RNAi technology. dNRAMP mutant flies were shown to be protected from viral infection (Rose et al., 2011). Interestingly, Carpenter et al. identified many differentially expressed genes in sigma virus-infected flies, several of which are neither up-regulated by bacterial or fungal infection, nor controlled by Toll, IMD, or JAK/STAT pathways, implying the involvement of other distinct regulatory immune mechanisms in defense against sigma virus in infected flies (Carpenter et al., 2009).

Ongoing studies are now applying genome-wide association study (GWAS) to identify the genetic basis of natural variation in *Drosophila's* immunity against pathogens. A study by Chapman et al. identified single nucleotide polymorphisms associated with genes (*Bomanin gene BomBc1, krishah*, and *S6k*) that significantly affected the fly's immunity to *Enterococcus faecalis* infection. Surprisingly, none of these genes are classified as canonical immune genes (Chapman et al., 2020).

Currently, the direction in unraveling host defense factors and innate immune effector molecules in the *Drosophila* model organism is heading toward bracketing classical genetic approaches with GWAS and genome-wide RNAi screening of flies with either loss of function or over-expressed immune genes, in addition to the use of co-immunoprecipitation assays and mass spectrometry to identify immune protein complexes. Moreover, bioinformatics analysis is being extensively adapted in deciphering candidate molecules and post-translational alterations that could impact the host's immune signaling pathways (Kanoh et al., 2019; Chapman et al., 2020). Nevertheless, and along with the ongoing high-throughput screens to discover conserved genes involved in host–pathogen interactions and immune signaling, the CRISPR/Cas9 technology has paved the way for additional wide-scale-based screens in *Drosophila* cultured cells, the results of which could be followed up *in vivo* in flies and/or mammals (Viswanatha et al., 2019). Some identified host factors required for defense against a broad spectrum of pathogens are summarized in Table 1.

**Table 1:** Host defense factors.

**Pathogen**	**Host**	**Defense factor**	**Lessons from *Drosophila***	**References**
*Bacillus thuringiensis* and *Erwinia carotovora carotovora*	Adult flies	Diuretic Hormone 31 (DH31)	The DH31 enteroendocrine peptide stimulates gut contractions, favoring the elimination of opportunistic bacteria	Benguettat et al., 2018
*Erwinia carotovora* *carotovora*	Adult flies	*Drosophila* Peroxiredoxin V (dPrxV)	*dPrxV* mutant flies exhibit reduced survival after gut infection The JNK/FOXO signaling mediated expression of the immune-related antioxidant enzyme dPrxV plausibly protects the host gut epithelial cells from oxidative damage during bacterial infection	Ahn et al., 2012
*Klebsiella pneumoniae*	Adult flies	Phg1	Phg1 is implicated in resistance to Klebsiella infection	Benghezal et al., 2006
*Photorhabdus asymbiotica* and *Photorhabdus luminescens*	Adult flies	PGRP-LE	*PGRP-LE* expression is upregulated following *Photorhabdus* infection Absence of functional *PGRP-LE* alters the transcriptional pathway activity of JNK and IMD signaling upon infection with *Photorhabdus asymbiotica*	Chevee et al., 2019
			*PGRP-LE* mutant flies are more sensitive to *Photorhabdus luminescens* *Photorhabdus luminescens* infection modifies the activity of JAK/STAT signaling	
*Scedosporium apiospermum* and *Scedosporium prolificans*	Adult flies	Toll Pathway	Wild-type flies are resistant to *Scedosporium apiospermum* and *Scedosporium* *prolificans* infections while Toll-deficiency results in acute infection and high mortality rates	Lamaris et al., 2007
*Cryptococcus* *neoformans*	Adult flies	Toll Pathway	The Toll pathway is necessary for clearing *Cryptococcus neoformans* introduced directly into the fly hemolymph and for the survival of systemically infected flies	Apidianakis et al., 2004
Zygomycetes	Adult flies	Toll Pathway	Zygomycetes rapidly infect and kill wild-type flies and *Toll*-deficient flies exhibit increased susceptibility to Zygomycetes	Chamilos et al., 2008
		*Eater*	Phagocytosis impaired *eater* mutant flies exhibit increased susceptibility to Zygomycetes infection	

## Pathogen Virulence Factors

Diverse well-designed screening assay systems have been established to identify virulence factors contributing to pathogen-induced host killing using the *Drosophila* model organism. Screening for virulence-attenuated mutants identified a set of genes involved in the multi-host pathogenesis of *P. aeruginosa* PA14, for example. Follow-up studies further characterized these genes to validate the use of *Drosophila* as a model for high-throughput identification of novel virulence factors. Characterizing *hudR*, an identified virulence gene encoding a MarR/SlyA family transcription factor, for instance, revealed that eminent expression of *hudA* (homologous to *UbiD*) is required and adequate to attenuate the virulence of hudR mutants in infected flies (Kim et al., 2008). Since quorum sensing is involved in the pathogenicity of *P. aeruginosa*, several studies focused on identifying quorum sensing-regulated virulence factors, as an appealing therapeutic approach to control *P. aeruginosa* infection (Bjarnsholt and Givskov, 2007). *P. aeruginosa* oxylipin lipids were also identified as pathogenic factors that promote bacterial virulence and biofilm formation in *Drosophila* (Martinez and Campos-Gomez, 2016). Further studies in *P. aeruginosa* also revealed that the phosphorylation state of the transcriptional response regulator AlgR inversely controls the production of pyoverdine and pyocyanin, two important *P. aeruginosa* virulence factors (Little et al., 2018). Other studies also focused on deciphering the effect of bacterial toxins on the host using fruit flies. *P. aeruginosa* exotoxin ExoS was shown to affect the activity of Rho GTPases, as the directed expression of the bacterial ExoS GAP domain (ExoSGAP) inhibits Rac1-, Cdc42-, and Rho-dependent signaling, suppressing *Drosophila* cellular immunity (Avet-Rochex et al., 2005). *V. cholerae* toxin, in turn, was shown to decrease intestinal stem cell division, alter epithelial regeneration, and induce cell–cell junctional damage (Guichard et al., 2013; Wang et al., 2013). Interestingly, the Vibrio polysaccharide (VPS)-dependent biofilm, which is highly activated upon entry into the arthropod intestine, is essential for *Drosophila* intestinal colonization (Purdy and Watnick, 2011). Surprisingly however, quorum sensing promotes a more auspicious interaction between the fly host and *V. cholerae* by reducing the nutritional burden of intestinal colonization in the host (Kamareddine et al., 2018b). Novel *H. pylori* effector proteins like the cytotoxin-associated gene A (CagA) have been studied in transgenic *Drosophila* flies. CagA mimics the eukaryotic adaptor protein Grb2-associated binder (Gab) and activates phosphatase SHP-2, a component of the receptor tyrosine kinase pathways. These findings in *D. melanogaster* could provide more insight into the role of translocated bacterial proteins that targets highly conserved eukaryotic cellular processes (Botham et al., 2008). The Anthrax toxin produced by *Bacillus anthracis* is comprised of protective antigen (PA), edema factor (EF), and lethal factor (LF) (Lacy and Collier, 2002). Similar to their function in mammals, LF cleaves MAPK kinases, and EF inhibits hedgehog pathway in flies (Guichard et al., 2006). This similarity in function strengthens the argument of choosing *Drosophila* as a multicellular host system to study *in vivo* function of virulence factors and diverse toxins. *Drosophila* has been also extensively used to study infectious properties of several bacterial species like *Porphyromonas gingivalis* (W83), a gram-negative obligate anaerobic bacteria strongly implicated in adult periodontitis (Griffen et al., 1998; Ezzo and Cutler, 2003; Igboin et al., 2011). *P. gingivalis* causes systemic infection in *Drosophila* and promotes potent killing in a dose-dependent manner. Interestingly, both heat-killed and live *P. gingivalis* are similarly pathogenic to the fly, suggesting a role of *P. gingivalis* cell surface components and *Drosophila* immunity in dictating pathology in this host–pathogen model (Igboin et al., 2011). Tabuchi et al. demonstrated an important role of *dltA*, a gene responsible for D-alanylation of techoic acid in the cell wall of gram-positive *S. aureus*, in inhibiting the fly's Toll pathway. *S. aureus*-infected *dltA* mutant flies exhibited an increase in life span compared to flies expressing *dltA* normally (Tabuchi et al., 2010). Fungal virulence factors have been also reported in several *Drosophila* studies. Gliotoxin, for example, contributes to the virulence of *Aspergillus fumigatus* in fruit flies with functional phagocytes as well as in non-neutropenic mice, suggesting that gliotoxin principally targets neutrophils or other phagocytes (Spikes et al., 2008). Cas5 transcription in *Candida albicans* regulates cell wall integrity and is essential for fungal virulence in both murine and Toll mutant flies (Chamilos et al., 2009). Likewise, *Candida glabrata* mutant strains lacking the yapsin virulence factors or the high-osmolarity glycerol pathway exhibit a less virulent effect in infected flies (Quintin et al., 2013). Several antifungal drug efficacy studies against invasive aspergillosis (Lionakis et al., 2005) and malasseziosis (Merkel et al., 2018) have been conducted in the *Drosophila* model. Many studies have also demonstrated the ability of parasitic nematodes, like those belonging to the *Heterorhabditis* genus, to infect and kill fruit flies at larval and adult stages and to trigger an up-regulation in several genes belonging to the Toll, IMD, JAK/STAT, and TGF-β signaling pathways (Castillo et al., 2013, 2015; Arefin et al., 2014). *Heterorhabditis gerrardi*, for example, harbors the pathogenic *Photorhabdus asymbiotica* bacteria, which gets ejected from the nematode gut into the host's hemolymph. In the hemolymph, *Photorhabdus asymbiotica* proliferates and releases toxins and virulence factors that kills the host and provides a favorable environment for the nematode (Waterfield et al., 2008; Eleftherianos et al., 2010). To analyze the impact of the *Photorhabdus-Heterorhabditis* mutualistic relation on the transcriptional profiles of the host, Castillo et al. (2015) performed next-generation RNA-sequencing on flies infected with *Photorhabdus* alone, germ-free *Heterorhabditis* lacking *Photorhabdus*, and *Heterorhabditis* carrying *Photorhabdus*. The bioinformatics analysis of that study revealed an impact of *Photorhabdus* on the transcription of fly genes associated with translational repression and stress responses, and an effect of *Heterorhabditis* on the expression profiles of genes involved in metabolism, lipid homeostasis, stress responses, DNA/protein synthesis, and functions of the nervous system.

In the last few years, a number of studies have examined virulence factors of human viral pathogens in *Drosophila*, as the fruit fly model facilitates the implementation of systematic, genome-wide RNAi analysis commonly used to identify genes that are involved in viral replication (Kuttenkeuler and Boutros, 2004). Since a broad spectrum of RNA viruses exploit internal ribosome entry sites (IRESs) for translation, genome-wide RNAi screen in *Drosophila* cells infected with *Drosophila* C virus were performed to reveal host factors required for IRES-dependent translation and viral replication. A study by Cherry et al. revealed 66 ribosomal proteins needed for *Drosophila* C virus, but not for non-IRES-containing RNA virus (Cherry et al., 2005). Some identified pathogen virulence factors are summarized in Table 2.

**Table 2:** Pathogen Virulence Factors.

**Pathogen**	**Host**	**Virulence factor**	**Lessons from *Drosophila***	**References**
*Pseudomonas aeruginosa*	Adult flies	Cyanide	Cyanogenic *Pseudomonas aeruginosa* strains cause motionlessness and bradycardia and contribute to lethality in infected flies	Broderick et al., 2008
	Adult flies and larvae	ExoS exotoxin	ExoSGAP acts as a negative regulator of RhoGTPases Rac1, Rho1 and Cdc42 in the fly eye/during eye morphogenesis	Avet-Rochex et al., 2005
	Adult flies	*FprA*	Role of *FprA* gene in superoxide-mediated stress protection and virulence of *Pseudomonas aeruginosa*	Boonma et al., 2017
	Adult flies	Ribonucleotide reductases (RNRs)	RNRs contribute to *Pseudomonas aeruginosa* pathogenicity in infected flies	Sjoberg and Torrents, 2011
	Adult flies	*pilGHIJKL chpABCDE* (*pil chp* gene cluster)	The *pilGHIJKL chpABCDE* gene cluster is required for twitching motility and potentially encodes a signal transduction system that controls the expression of virulence factors	D'Argenio et al., 2001
	Adult flies	*RelA*	*RelA* plays a role in bacterial adaptation to nutritional deficiencies by the production of guanosine pentaphosphate or tetraphosphate *Pseudomonas aeruginosa* strains lacking *relA* demonstrate reduced virulence in *Drosophila melanogaster* feeding assay	Erickson et al., 2004
*Helicobacter pylori*	Adult flies, larvae, and pupae	Cytotoxin associated gene A (CagA) protein	CagA mimics the eukaryotic Grb2-associated binder (Gab) adaptor protein and activates SHP-2, a component of receptor tyrosine kinase (RTK) pathways	Botham et al., 2008
	Adult flies		CagA promotes microbial dysbiosis and exacerbates epithelial cell proliferation	Jones et al., 2017
*Bacillus anthracis*	Adult flies and larvae	Toxin Lethal factor (LF) and edema factor (EF)	LF and EF cooperatively inhibit endocytic recycling by the Rab11/Sec15 exocyst	Guichard et al., 2010
*Erwinia carotovora*	Larvae	Erwinia Virulence Factor (Evf)	Evf promotes accumulation of bacteria inside the larval gut, affecting the gut physiology	Acosta Muniz et al., 2007
*Vibrio cholera, Yersinia pseudotuberculosis*, and *Pseudomonas aeruginosa*	Adult flies	KerV virulence factor	KerV plays an important role in the *Vibrio cholera, Yersinia pseudotuberculosis*, and *Pseudomonas aeruginosa*	An et al., 2009
*Staphylococcus aureus*	Adult flies	Wall teichoic acids (WTA)	WTA promotes bacterial pathogenicity by limiting the ability of PGRP-SA to recognize *Staphylococcus aureus*	Atilano et al., 2011
*Mycobacterium marinum*	Adults flies	*Mag24*	Absence of *mag24* gene attenuates the virulence of *Mycobacterium marinum*	Dionne et al., 2003
*Candida albicans*	Adult flies	Efg1p and Cph1p	*cph1/cph1* and *efg1/efg1 C. albicans* mutants have attenuated virulence, and *efg1/efg1 cph1/cph1* double mutants are almost avirulent in *Toll* deficient flies	Chamilos et al., 2006
*Aspergillus fumigatus*	Adult flies	*Alb1*	*Toll*-deficient *Drosophila* infected with *alb1*-deleted hypovirulent *Aspergillus* mutant survives better than those infected with a wild-type *Aspergillus* strain	Lionakis et al., 2005
*Cryptococcus neoformans*	Adult flies	Protein kinase A and RAS signal transduction pathways	Protein kinase A (PKA) and RAS signal transduction pathways in *Cryptococcus neoformans* are involved in *Drosophila* killing	Apidianakis et al., 2004

## Conclusion

The use of animal models serves as a foundation to reveal conserved aspects of human disease. Unraveling detailed mechanisms of host–pathogen interactions using *Drosophila* provides further insight into the pathogenic arm of a microorganism and the defensive arm of a host. A better understanding of host–microbe interactions is desirable for the development of novel successful treatment regimens for pathogen-caused diseases.

## Author Contributions

All authors listed have made a substantial, direct and intellectual contribution to the work, and approved it for publication.

## Conflict of Interest

The authors declare that the research was conducted in the absence of any commercial or financial relationships that could be construed as a potential conflict of interest.
